# Twenty-four-hour National Institute of Health Stroke Scale predicts short- and long-term outcomes of basilar artery occlusion after endovascular treatment

**DOI:** 10.3389/fnagi.2022.941034

**Published:** 2022-10-20

**Authors:** Jing Chen, Shuai Liu, Mingchao Wu, Ling Dai, Jie Wang, Weihua Xie, Yuqi Peng, Jinlin Mu, Shunyu Yang, Jinbo Ran, Jian Zhang, Wenshu Niu, Jingbang Zheng, Junxiong Wu, Guangxiong Yuan

**Affiliations:** ^1^Department of Neurology, Baoji Central Hospital, Baoji, Shanxi, China; ^2^Department of Neurology, Xinqiao Hospital and the Second Affiliated Hospital, Army Medical University (Third Military Medical University), Chongqing, China; ^3^Department of Neurology, Jingdezhen No.1 People’s Hospital, Jingdezhen, Jiangxi, China; ^4^Department of Neurology, Luxian People’s Hospital, Luzhou, Sichuan, China; ^5^Department of Neurology, Chongqing Traditional Chinese Medicine Hospital, Chongqing, China; ^6^Department of Neurology, People’s Hospital of Mengzi, Mengzi, Yunnan, China; ^7^Center of Brain, Sichuan Science City Hospital, Mianyang, China; ^8^Department of Neurology, Traditional Chinese Medicine Hospital of Nanjiang, Bazhong, Sichuan, China; ^9^Department of Neurology, The First People’s Hospital of Yunnan, Kunming, Yunnan, China; ^10^Department of Neurology, People’s Hospital of Dejiang, Tongren, Guizhou, China; ^11^Department of Neurology, The Second Affiliated Hospital of Guangxi Medical University, Nanning, Guangxi, China; ^12^Department of Neurology, The 988th Hospital of the People’s Liberation Army, Zhengzhou, Henan, China; ^13^Department of Neurology, Chongqing Sanbo Changan Hospital, Chongqing, China; ^14^Department of Emergency, Xiangtan Central Hospital, Xiangtan, Hunan, China

**Keywords:** stroke, basilar, National Institute of Health Stroke Scale (NIHSS), endovascular treatment, outcome

## Abstract

**Background:**

The present study aimed to evaluate the prognostic value of the 24-h National Institute of Health Stroke Scale (NIHSS) for short- and long-term outcomes of patients with basilar artery occlusion (BAO) after endovascular treatment (EVT) in daily clinical routine.

**Methods:**

Patients with EVT for acute basilar artery occlusion study registry with the 24-h NIHSS, and clinical outcomes documented at 90 days and 1 year were included. The NIHSS admission, 24-h NIHSS, NIHSS delta, and NIHSS percentage change, binary definitions of early neurological improvement [ENI; improvement of 4/(common ENI)/8 (major ENI)/10 (dramatic ENI)] NIHSS points were compared to predict the favorable outcomes and mortality at 90 days and 1 year. The primary outcome was defined as favorable if the modified Rankin Scale (mRS) score was 0–3 at 90 days.

**Results:**

Of the 644 patients treated with EVT, the 24-h NIHSS had the highest discriminative ability for favorable outcome prediction [receiver operator characteristic (ROC)_NIHSS 24 h_ area under the curve (AUC): 0.92 (0.90–0.94)] at 90 days and 1 year [(ROC_NIHSS 24 h_ AUC: 0.91 (0.89–0.94)] in comparison to the NIHSS score at admission [ROC_NIHSS admission_ AUC at 90 days: 0.73 (0.69–0.77); 1 year: 0.74 (0.70–0.78)], NIHSS delta [ROC_Δ NIHSS_ AUC at 90 days: 0.84 (0.81–0.87); 1 year: 0.81 (0.77–0.84)], and NIHSS percentage change [ROC_%change_ AUC at 90 days: 0.85 (0.82–0.89); 1 year: 0.82 (0.78–0.86)].

**Conclusion:**

The 24-h NIHSS with a threshold of ≤23 points was the best surrogate for short- and long-term outcomes after EVT for BAO in the clinical routine.

## Introduction

Basilar artery occlusion (BAO) is rare and accounts for approximately 1% of all strokes (Archer and Horenstein, 1977; Mattle et al., 2011). Despite progress in the management of acute stroke, almost 70% of patients die or remain severely disabled (Schonewille et al., 2009; Singer et al., 2015). Therefore, early prognosis of short- and long-term outcomes in patients with BAO can facilitate medical decision-making.

Various definitions of early neurological improvement (ENI) based on the National Institute of Health Stroke Scale (NIHSS) have been widely used to predict short-term favorable outcomes in anterior circulation and hemispheric strokes after endovascular treatment (EVT) (Meyer et al., 2020). However, early prognosis of clinical outcomes following BAO remains unclear. A subgroup analysis of the Basilar Artery International Cooperation Study (BASICS) registry revealed that the 24- to 48-h NIHSS predicts poor outcomes and mortality at 1 month. However, other indexes of ENI have not been considered together to investigate the best surrogates for outcome prediction in patients with BAO. In addition, only 1-month outcomes were evaluated in that study; short- and long-term outcomes such as a modified Rankin Scale (mRS) of 0–3 at 90 days and 1 year were not evaluated. Thus, there is an urgent need to evaluate early clinical surrogates for predicting short- and long-term clinical outcomes after EVT in daily clinical routine.

Early neurological examination, such as the NIHSS score at 24 h and relative change in the NIHSS score, reflect *both the severity of neurological deficits at admission as well as* the effects of EVT (Frankel et al., 2000; Saver and Altman, 2012). Early improvement may be a symbol of recanalization of BAO, whereas the failure of improvement or clinical decline may signify futile recanalization or medical complications. Although individual characteristics of patients at admission can facilitate medical decision-making, the prognosis of short- and long-term functional favorable outcomes and the decision to prolong life-sustaining medical and surgical treatments usually occur in the first 24 h (Rangaraju et al., 2016). Therefore, early clinical surrogates, especially the early index after EVT, may predict short- and long-term outcomes in patients with BAO.

In this study, we aimed to evaluate early clinical surrogates together in patients with BAO after EVT in a daily clinical routine to better estimate the odds for short- and long-term clinical outcomes and to improve medical decision-making.

## Materials and methods

### Study design and data sources

We used data from the BASILAR registry,^1^ which is a nationwide prospective registry and includes 47 Chinese comprehensive stroke centers and a pool of individual data of consecutive adult patients for whom EVT was indicated for acute BAO within 24 h of symptom onset. The study protocol was approved by the ethics committee of each center. Written informed consent was obtained from all the patients or their legally authorized representatives. Patients with an NIHSS score documented at 24 h were included in this study.

### Early neurological improvement

Two binary definitions of ENI were applied based on the NIHSS at 24 h after EVT, as previously described: (1) NIHSS improvement ≥4 points from baseline (common ENI) (Song et al., 2015), (2) NIHSS improvement ≥8 points from baseline (major ENI) (Broderick et al., 2000; Brown et al., 2004), and (3) NIHSS improvement ≥10 points from baseline (dramatic ENI) (Meyer et al., 2020). We calculated the cut-off values for the NIHSS score at admission, the NIHSS score at 24 h, absolute NIHSS change (ΔNIHSS), and NIHSS percentage change [% NIHSS: (NIHSS admission − NIHSS 24 h) / NIHSS admission] with the predictive values of highest sensitivity and specificity (Youden index) for a favorable functional outcome (mRS 0–3) and mortality at 90-day and 1-year follow-up using receiver operator characteristics (ROCs). The area under the curve (AUC) values of 24-h NIHSS were compared with other indexes using the DeLong method (DeLong et al., 1988). The AUC and 95% confidence interval (CI) of NIHSS at 24 h after EVT were evaluated in patients stratified by the type of anesthesia and intubation. Furthermore, the AUC and 95% CI of all indexes were compared by stratification based on different levels of stroke severity (mild: NIHSS 0–9; moderate: NIHSS 10–20; severe: ≥21). Independent predictors for reaching the cut-off value with the highest value for predicting 90-day favorable outcomes were subsequently identified using a multivariable logistic analysis in the study cohort. Subgroup analysis was performed to identify factors that significantly affected the prediction reversion in patients with poor outcomes at 90 days, despite reaching the threshold at 24 h.

### Outcome measurement

The primary endpoint was a functional favorable outcome defined as a mRS of 0–3 at 90 days. Secondary outcomes included favorable outcomes at 1 year and all-cause mortality at 90 days and 1 year. Successful recanalization was defined as a modified thrombolysis in cerebral infarction (mTICI) score of 2b or 3, which was confirmed by catheter angiography at the end of the procedure (Zaidat et al., 2013). Intracerebral hemorrhage was assessed according to the Heidelberg Bleeding Classification (von Kummer et al., 2015).

### Statistical analyses

Data were presented as medians (interquartile ranges) or numbers with percentages. Scatterplots were used to assess the relationship between the 24-h NIHSS and favorable outcomes at 90 days and 1 year. The association of 24-h NIHSS scores with outcomes at different times was depicted in adjusted margin plots. Differences were considered significant at a *P*-value < 0.05, and all tests of hypotheses were two-sided. We excluded patients with missing essential data rather than imputation. SPSS version 23 (IBM Corp., Armonk, NY, USA), R version 3.2 (R Foundation for Statistical Computing, Vienna, Austria), and MedCalc (MedCalc Software Ltd., Belgium, Ostend, Belgium) were used for conducting the statistical analyses. A two-tailed *P*-value < 0.05 was considered statistically significant for all analyses.

## Results

### Study population

Of the 829 patients enrolled in BASILAR, 644 in the EVT group with an NIHSS score documented at 24 h, as well as a mRS and mortality at 90 days, were included in our analysis. Overall, age was a median of 64 [interquartile (IQR) 56–73] years, NIHSS was 27 (IQR 17–33), and pc-ASPECTS was 8 (IQR 7–9). In addition, 612 patients had a long-term outcome at 1 year. The baseline characteristics are shown in Table 1.

**TABLE 1:** Description of the study cohort.

Characteristics	Favorable outcome (*n* = 206)	Unfavorable outcome (*n* = 438)	*P*-value
Age, median (IQR), year	63 (55–71)	65 (57–74)	0.03
Men, *n* (%)	150 (72.8)	331 (75.6)	0.45
Admission NIHSS, median (IQR)	18 (10–27)	30 (21–34)	<0.001
Admission pc-ASPECTS, median (IQR)	9 (8–10)	8 (6–9)	<0.001
Intubation at presentation, *n* (%)	6 (2.91)	16 (3.65)	0.63
**History, *n* (%)**			
Hypertension	141 (68.4)	310 (70.8)	0.55
Hyperlipidemia	73 (35.4)	140 (32.0)	0.38
Diabetes mellitus	33 (16.0)	115 (26.3)	0.004
Atrial fibrillation	48 (23.3)	88 (20.1)	0.35
Coronary heart disease	23 (11.2)	81 (18.5)	0.018
Ischemic stroke	36 (17.5)	104 (23.7)	0.072
Cause of stroke, *n* (%)			0.10
Large artery atherosclerosis	121 (58.7)	295 (67.4)	
Cardioembolism	64 (31.1)	109 (24.9)	
Other causes	21 (10.2)	34 (7.8)	
Location of occlusion, *n* (%)			0.017
Distal basilar artery	88 (42.7)	132 (30.1)	
Middle basilar artery	54 (26.2)	141 (32.2)	
Proximal basilar artery	32 (15.5)	75 (17.1)	
Vertebral artery-V4	32 (15.5)	90 (20.5)	
Intravenous thrombolysis, *n* (%)	39 (18.9)	79 (18.0)	0.78
OPT, median (IQR), min	302 (202–433)	340 (234–505)	0.019
PTR, median (IQR), min	86 (61–128)	113 (79–158)	<0.001
Intubation at 24 h, *n* (%)	15 (7.3)	108 (24.7)	NA
**Severe adverse events, *n*/total *n* (%)**			
Pulmonary infection	115 (17.9)	365 (83.3)	<0.001
Gastrointestinal bleeding	20 (9.7)	94 (21.5)	<0.001
SICH	3 (1.5)	42 (9.8)	<0.001
24-h NIHSS	8 (3–16)	32 (27–36)	<0.001
ΔNIHSS_24 h_	5 (0–14)	−1 (−5 to 0)	<0.001
NIHSS percent change	38 (0–72.75)	−3.5 (−20 to 0)	<0.001
mTICI 2b-3	193 (93.7)	326 (74.4)	<0.001

IQR, interquartile range; NIHSS, National Institutes of Health Stroke Scale; pc-ASPECTS, Posterior Circulation-Alberta Stroke Program Early CT Score; OPT, onset-to-puncture time; PTR, puncture-to-revascularization time; SICH, symptomatic intracranial hemorrhage; mTICI, modified treatment in cerebral infarction; NA, not applicable.

### National Institute of Health Stroke Scale at 24 h strongly predicts favorable functional outcomes at 90 days and 1 year

**FIGURE 1:**
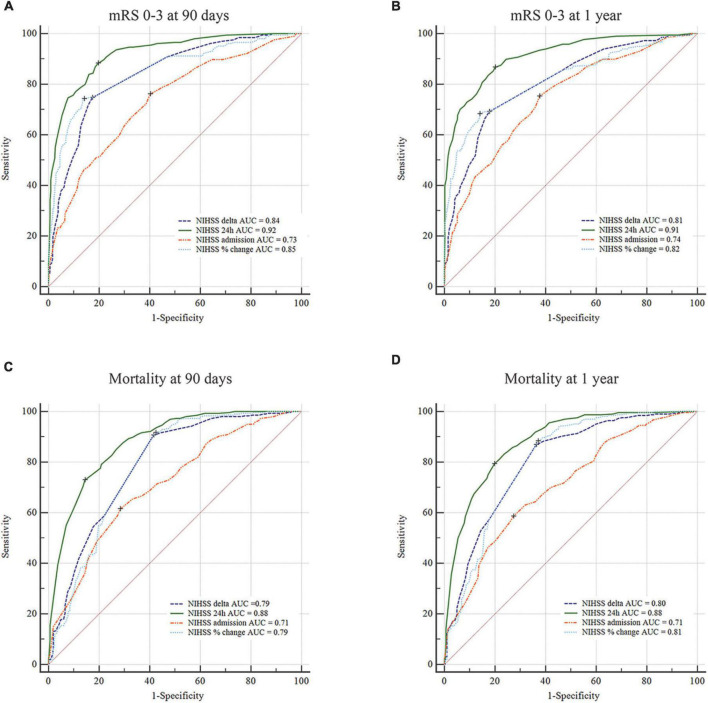
The National Institute of Health Stroke Scale (NIHSS) at 24 h predicts short- and long-term clinical outcomes in patients with basilar artery occlusion. **(A–D)** Comparison of the NIHSS at 24 h and other indexes based on the receiver operator characteristic (ROC) curves for predicting favorable functional outcome (mRS 0–3) and mortality at 90 days and 1 year.

The analysis of the entire study cohort revealed that the NIHSS at 24 h was significantly correlated with favorable outcomes at 90 days and 1 year (Figures 1A,B and Supplementary Figure 1). The ROC and AUC (95% CI) showed that the NIHSS at 24 h had the highest discriminative ability to predict favorable functional outcome at 90 days [ROC_NIHSS 24 h_, 0.92 (0.90–0.94)] and 1 year [ROC_NIHSS 24 h_, 0.91 (0.89–0.94)] in comparison to NIHSS at admission [ROC_NIHSS admission_, 90-day mRS 0–3: 0.73 (0.69–0.77), 1-year mRS 0–3: 0.74 (0.70–0.78)], ΔNIHSS [ROC_Δ NIHSS_, 90-day mRS 0–3: 0.84 (0.81–0.87), 1-year mRS 0–3: 0.81 (0.77–0.84)], and NIHSS percentage change [ROC_% change_, 90-day mRS 0–3: 0.85 (0.82–0.89), 1-year mRS 0–3: 0.82 (0.78–0.86)]. On comparing both discriminators with the highest AUCs, it was observed that the NIHSS at 24 h was significantly better than the NIHSS percentage change in favorable outcome prediction at 90 days (*P* < 0.001) and 1 year (*P* < 0.001). The NIHSS at 24 h was also superior to baseline NIHSS (90-day mRS 0–3: *P* < 0.001; 1-year mRS: *P* < 0.001) and ΔNIHSS (90-day mRS 0–3: *P* < 0.001; 1-year mRS: *P* < 0.001). At the maximum Youden index, the best cut-off point of the 24-h NIHSS was 23, with a sensitivity of 0.88 and a specificity of 0.80, in terms of 90-day favorable outcome prediction; for the 1-year prediction, the best cut-off point of the 24-h NIHSS was 25 with a sensitivity of 0.87 and a sensitivity of 0.80. Supplementary Figure 2A illustrates the predicted probabilities of favorable outcomes at 90 days and 1 year, with the 24-h NIHSS increment as a continuous variable.

### National Institute of Health Stroke Scale at 24 h strongly predicts mortality at 90 days and 1 year

As seen in Figures 1C,D, the AUC curves revealed that the 24-h NIHSS had the highest discriminative ability to predict mortality at 90 days [ROC_NIHSS 24 h_, 0.88 (0.85–0.90)] and 1 year [ROC_NIHSS 24 h_, 0.88 (0.85–0.91)] in comparison to the NIHSS scores at admission [ROC_NIHSS admission_, 90-day mortality: 0.71 (0.67–0.74), 1-year mortality: 0.71 (0.67–0.74)], ΔNIHSS [ROC_Δ NIHSS_, 90-day mortality: 0.79 (0.76–0.82), 1-year mortality: 0.80 (0.77–0.84)] and NIHSS percentage change [ROC_% change_, 90-day mortality: 0.79 (0.75–0.82), 1-year mortality: 0.81 (0.77–0.84)]. The NIHSS score at 24 h was significantly better than ΔNIHSS in predicting 90-day mortality (*P* < 0.001) and 1-year mortality (*P* < 0.001) and also better than NIHSS percentage change (90-day mortality: *P* < 0.001; 1-year mortality: *P* < 0.001). At 24 h, the optimal threshold of NIHSS for mortality prediction was 30 at 90 days (sensitivity: 0.73; specificity: 0.86) and 27 at 1 year (sensitivity: 0.79; specificity: 0.80). Supplementary Figure 2B illustrates the predicted probabilities of mortality at 90 days and 1 year, with the 24-h NIHSS increment as a continuous variable.

### Binary threshold

**FIGURE 2:**
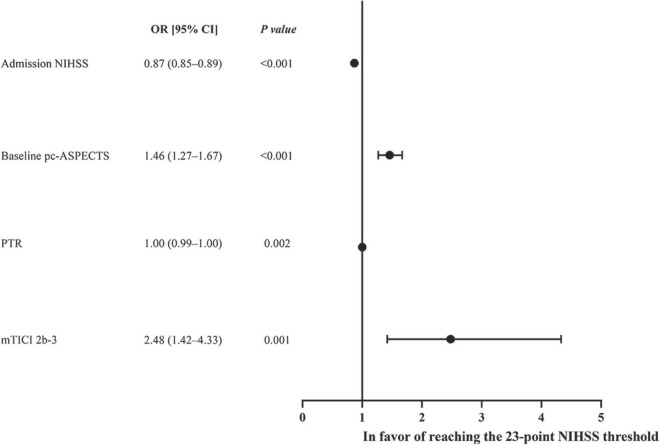
Forest plot of logistic regression anlysis for reaching the 24-h NIHSS threshold of 23 points.

**FIGURE 3:**
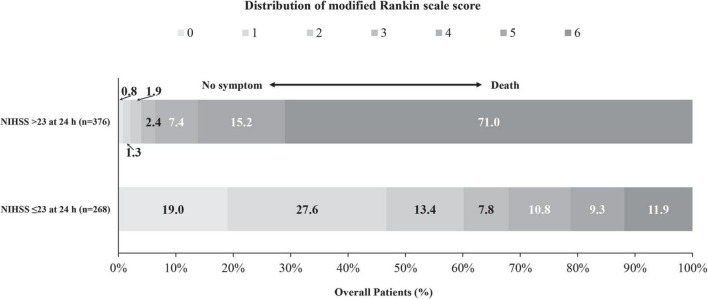
Distribution of the mRS scores at 90 days stratified by patients with and without reaching the ENI threshold of 23-point at 24 h after EVT.

The 23-point NIHSS threshold at 24 h after EVT identified 67.8% of patients with a mRS score of 0–3 in this subgroup (Figure 2). The multivariable logistic analysis revealed that the lower NIHSS at admission [adjusted odds ratio (OR): 0.87 (0.85–0.89), *P* < 0.001], higher pc-ASPECTS [adjusted OR: 1.46 (1.27–1.67), *P* < 0.001], shorter puncture-to-recanalization time [adjusted OR: 1.00 (0.99–1.00), *P* = 0.002], and mTICI 2b-3 [adjusted OR: 2.48 (1.42–4.33), *P* = 0.001] were significantly associated with reaching the 23-point NIHSS threshold (Figure 3). In contrast, within this subpopulation, multivariable logistic regression analysis revealed that advanced age [adjusted OR: 0.96 (0.94–0.99), *P* = 0.003], male sex [adjusted OR: 0.48 (0.24–0.97), *P* = 0.039], pulmonary infection [adjusted OR: 0.21 (0.11–0.43), *P* < 0.001], and gastrointestinal bleeding [adjusted OR: 0.43 (0.21–0.88), *P* = 0.022] were independent predictors that conferred significant impacts on turning the outcome prognosis from favorable to poor at 90 days (Table 2).

**TABLE 2:** Factors associated with poor outcome in subgroup reaching the 23-point NIHSS threshold at 24 h.

	Adjusted OR	95% CI	*P*-value
Age	0.96	0.94–0.99	0.003
Male	0.48	0.24–0.97	0.039
Pulmonary infection	0.21	0.11–0.43	<0.001
Gastrointestinal bleeding	0.43	0.21–0.88	0.022

### Sensitivity analyses

Sensitivity analyses were performed to evaluate the correlation of the ENI indexes with the clinical outcomes stratified by the type of anesthesia, intubation, and stroke severity. The NIHSS at 24 h showed similar prediction values for clinical outcomes in patients with different types of anesthesia (Supplementary Table 1). However, the AUC values of the 24-h NIHSS in patients with intubation were lower than those in patients without intubation for predicting clinical outcomes [AUC (95% CI), mRS 0–3 at 90 days: 0.87 (0.76–0.98) vs. 0.92 (0.90–0.94)]. In the analysis of patients stratified by stroke severity, the 24-h NIHSS outperformed baseline NIHSS, ΔNIHSS, NIHSS percentage change, cENI, dENI, and mENI in patients with moderate and severe stroke (Supplementary Table 2). In patients with mild stroke (NIHSS <10), the 24-h NIHSS, ΔNIHSS, and NIHSS percentage change showed similar AUC values in outcome prediction. In patients with moderate and severe stroke, the 24-h NIHSS outperformed other indexes.

## Discussion

To the best of our knowledge, this is the first study to systematically evaluate the prognostic value of early clinical surrogates in predicting 90-day and 1-year clinical outcomes in patients with BAO. Furthermore, our results highlight the importance of follow-up clinical information in the subacute stage rather than the information available at the time of admission. Our analysis illustrates that the NIHSS at 24 h (with a threshold of NIHSS ≤23) outperforms other early clinical surrogates, including baseline NIHSS, ΔNIHSS, and NIHSS percentage change, that predict 90-day and 1-year clinical outcomes, including favorable functional outcomes and mortality. In patients with intubation, the NIHSS at 24 h indicated lower discriminative ability to predict clinical outcomes than patients without intubation. In patients with a mild deficit (NIHSS <10), the 24-h NIHSS showed a similar discriminative ability to predict clinical outcomes as well as ΔNIHSS and NIHSS percentage change. In addition, a lower NIHSS score at admission, higher pc-ASPECTS, shorter puncture-to-recanalization time, and mTICI 2b-3 were significantly associated with reaching the 23-point NIHSS threshold. Advanced age, male sex, pulmonary infection, and gastrointestinal bleeding have a significant impact on reversing the prognosis from favorable to poor outcomes, despite reaching the 23-point threshold of NIHSS at 24 h after EVT.

According to the subgroup analysis of the BASICS trial, the 24- to 48-h NIHSS could be used to accurately predict the 1-month outcome and mortality (Rangaraju et al., 2016). This was the first study to demonstrate the high predictive power and potential clinical utility of the 24- to 48-h NIHSS in patients with BAO. However, this study did not use the 90-day time point in the literature available on stroke because the primary outcome measure was obtained at 1 month in the BASICS trial. Significant neurological recovery could occur between 3 and 12 months after BAO stroke, and several patients may attain functional independence at 90 days or later, even if they had some degree of disability at discharge or at 1 month. Our study revealed that the threshold of the 24-h NIHSS for predicting the favorable outcome at 1-year was widened than the threshold for predicting outcomes at 90 days. Nevertheless, the threshold for mortality prediction was narrower at 90 days than that at 1 year. These results suggest that some patients with a mild level of disability at 90 days could still attain favorable independence, provided that they underwent an aggressive rehabilitation program and that the risk factors such as coronary heart disease and atrial fibrillation were well controlled to avoid a heart attack or reocclusion (Wu et al., 2021).

In patients with BAO stroke, the NIHSS is well-accepted and widely used to evaluate neurological deficits (Greving et al., 2012; Heldner et al., 2013). The NIHSS score at admission often changes within the first 24 h after EVT (Saver and Altman, 2012). Rapid alleviation or even reversal of neurological deficits often represents successful mechanical thrombectomy or spontaneous recanalization (Hendrix et al., 2021). Clinical deterioration may indicate infarct expansion, stroke-related or intervention-related complications, futile reperfusion, or hemorrhage (Brown et al., 2004). However, some patients with recanalization exhibited a “stunned brain” phenomenon (delayed or no response to recanalization for a few hours) and can achieve significant neurological recovery at a later stage (Alexandrov et al., 2004). This might occur when patients are treated under general anesthesia, which can influence the levels of consciousness during the early neurological assessment. In our study, the 24-h NIHSS score was not influenced by anesthesia, and it was a good early surrogate to predict short- and long-term clinical outcomes. Previous studies found that the prediction value of 24-h NIHSS cannot be influenced by intubation (Rangaraju et al., 2016); however, in our analysis, we found that patients with intubation showed a lower discriminative ability to predict clinical outcomes than those without intubation. This could be explained by the fact that language/speech domains of the NIHSS could be influenced by intubation. Moreover, using the 24-h NIHSS as an early accurate prognostication tool can guide physicians and pursue or withdraw medical decisions in a timely manner.

In our study, a 23-point threshold NIHSS score at 24 h could identify 67.8% of patients with a mRS score of 0–3 in this subgroup. According to a subgroup analysis of BASICS, a 24-h NIHSS of 0–11 could identify 75% (114/152) of patients with a favorable outcome, whereas a 22-point threshold could identify only 55.4% (128/231) of patients with a favorable outcome (Rangaraju et al., 2016). This could be explained by the fact that severely affected patients were predominantly enrolled in the BASILAR registry compared with the BASICS registry. Because the anatomical characteristics of the posterior circulation differ from those of the anterior circulation, clinical symptoms differ. Clinical symptoms depend on the location of the occlusion and the anatomical regions influenced by the resulting ischemia. In the BASILAR registry, many patients with middle occlusion sites were enrolled, and they showed higher morbidity and mortality due to brain stem involvement (Tatu et al., 1996). Therefore, the threshold of 24-h NIHSS in our study was higher. Previous studies found that a baseline NIHSS score ≥13 precited poor functional outcomes in patients with BAO (Gory et al., 2018). However, another study illustrated that a baseline NIHSS score ≥18 was the threshold for poor outcomes (Linfante et al., 2016). However, there were few studies identifying the cut-off score based on the 24-h NIHSS, in which EVT for BAO could be indicated. Hence, a larger cohort of BAO is required to explore the cut-off score based on the NIHSS. In our cohort, 32% (86/268) of the patients had a poor outcome despite reaching the threshold at 24 h. Accordingly, we identified that factors such as advanced age and male sex, gastrointestinal bleeding, and pulmonary infection were independent predictors for the reversion of the outcome prediction at 90 days in this subpopulation. This finding was consistent with the previous studies, which revealed that these factors were associated with poor outcomes (Kent et al., 2005; O’Donnell et al., 2008; Luo et al., 2021). In future, the prognostication of NIHSS at 24 h needs to be verified in larger cohorts of BAO. In addition, the threshold of 24-h NIHSS should also be validated in the external cohort.

Our study has several limitations, including the fact that the study design was derived from a prospective registry. In addition, we did not consider the effects of psychological and socioeconomic factors that may influence stroke outcomes. Also, the mRS does not represent the quality of life after stroke. Moreover, we did not evaluate the clinical and imaging variables, such as age, successful recanalization, pc-ASPECTS, or extensive brainstem infarction in the imaging study. The NIHSS cannot capture gait, mobility measures, and cognition measures, such as visuospatial and executive functions. Thus, the NIHSS score evaluation method for these patients is limited. Finally, the results must be interpreted with caution because of the limits related to the high cut-off 24-h NIHSS, and a threshold of 23 may have limited clinical value. Confirmatory randomized trials in these patient populations are desirable.

## Conclusion

In conclusion, this study suggests that the 24-h NIHSS with a threshold of ≤23 points strongly predicts short- and long-term clinical outcomes after EVT in BAO. In addition, patients with a lower NIHSS score at admission, higher pc-ASPECTS, shorter puncture-to-recanalization time, and mTICI 2b-3 were more likely to reach the 23-point NIHSS threshold. Nevertheless, advanced age, male sex, pulmonary infection, and gastrointestinal bleeding have a significant impact on reversing the prognosis from favorable to poor outcomes, despite reaching the 23-point threshold of NIHSS at 24 h after EVT.

## Data availability statement

The original contributions presented in this study are included in the article/Supplementary material, further inquiries can be directed to the corresponding authors.

## Ethics statement

The studies involving human participants were reviewed and approved by the Xinqiao Hospital Ethics Committee. The patients/participants provided their written informed consent to participate in this study.

## Author contributions

JC and SL were involved in statistical analysis, study design, and manuscript draft. MW, LD, JW, WX, YP, JM, SY, JR, JZ, WN, and JBZ were involved in data collection. JXW and GY were involved in study design, manuscript editing, and final approval of this manuscript. All authors contributed to the article and approved the submitted version.

## Footnotes

1 http://www.chictr.org.cn/, Identifier: ChiCTR1800014759.
