# Supplementary material for: Twenty-four-hour National Institute of Health Stroke Scale predicts short- and long-term outcomes of basilar artery occlusion after endovascular treatment
Source: Front Aging Neurosci. 2022 Oct 20;14:941034. doi: 10.3389/fnagi.2022.941034 (PMC9632169; doi:10.3389/fnagi.2022.941034)
Supplement: Supplementary file 1 [file Data_Sheet_1.docx]

**eTable 1.** ROC-based analysis of 24-h NIHSS for clinical outcomes by type of anesthesia and intubation

| Study cohort and subgroups | Area under the curve values and 95% CI for outcomes at different times | | | |
| --- | --- | --- | --- | --- |
|  | 90-day outcome (n=640) | | 1-year outcome (n=608) | |
|  | mRS 0–3 | mortality | mRS 0–3 | mortality |
| Type of anesthesia |  |  |  |  |
| General anesthesia | 0.92 (0.88–0.96) | 0.87 (0.83–0.91) | 0.88 (0.84–0.92) | 0.87 (0.82–0.91) |
| Conscious sedation | 0.93 (0.89–0.98) | 0.89 (0.84–0.94) | 0.93 (0.88–0.98) | 0.91 (0.87–0.95) |
| Local anesthesia | 0.92 (0.89–0.95) | 0.89 (0.84–0.93) | 0.94 (0.90–0.97) | 0.86 (0.80–0.93) |
| Intubation at 24 h |  |  |  |  |
| Yes | 0.87 (0.76–0.98) | 0.77 (0.69–0.86) | 0.84 (0.76–0.93) | 0.84 (0.76–0.92) |
| No | 0.92 (0.90–0.94) | 0.89 (0.87–0.92) | 0.92 (0.90–0.95) | 0.89 (0.86–0.92) |

**eTable 2.** ROC-based analysis of different surrogates for clinical outcomes by stroke severity

| Study cohort and subgroups | Surrogates | Area under the curve values and 95% CI for outcomes at different times | | | |
| --- | --- | --- | --- | --- | --- |
|  |  | 90-day outcome (n=640) | | 1-year outcome (n=608) | |
|  |  | mRS 0–3 | mortality | mRS 0–3 | mortality |
| NIHSS 0–9 | Admission NIHSS | 0.72 (0.58–0.85) | 0.66 (0.50–0.82) | 0.66 (0.50–0.81) | 0.65 (0.48–0.81) |
|  | 24-h NIHSS | 0.94 (0.87–1.00) | 0.93 (0.86–0.99) | 0.93 (0.84–1.00) | 0.90 (0.79–1.00) |
|  | △NIHSS 24h | 0.95 (0.91–1.00) | 0.94 (0.88–0.99) | 0.97 (0.93–1.00) | 0.95 (0.87–1.00) |
|  | NIHSS percent change | 0.95 (0.91–1.00) | 0.94 (0.88–1.00) | 0.97 (0.93–1.00) | 0.94 (0.88–1.00) |
|  | cENI | 0.87 (0.75–0.98) | 0.89 (0.77–1.00) | 0.91 (0.81–1.00) | 0.88 (0.76–1.00) |
| NIHSS 10–20 | Admission NIHSS | 0.60 (0.51–0.69) | 0.59 (0.49–0.69) | 0.60 (0.51–0.70) | 0.60 (0.51–0.70) |
|  | 24-h NIHSS | 0.92 (0.88–0.96) | 0.90 (0.85–0.95) | 0.90 (0.85–0.95) | 0.90 (0.85–0.95) |
|  | △NIHSS 24h | 0.90 (0.86–0.95) | 0.87 (0.81–0.93) | 0.87 (0.82–0.93) | 0.87 (0.82–0.93) |
|  | NIHSS percent change | 0.91 (0.86–0.96) | 0.87 (0.82–0.93) | 0.88 (0.82–0.93) | 0.88 (0.82–0.94) |
|  | cENI | 0.77 (0.69–0.85) | 0.79 (0.70–0.87) | 0.77 (0.69–0.85) | 0.78 (0.70–0.87) |
|  | dENI | 0.66 (0.57–0.76) | 0.62 (0.53–0.71) | 0.66 (0.56–0.75) | 0.64 (0.55–0.73) |
|  | mENI | 0.65 (0.56–0.74) | 0.61 (0.52–0.70) | 0.65 (0.56–0.74) | 0.63 (0.54–0.73) |
| NIHSS 21–42 | Admission NIHSS | 0.65 (0.58–0.71) | 0.66 (0.61–0.71) | 0.67 (0.61–0.73) | 0.67 (0.61–0.72) |
|  | 24-h NIHSS | 0.90 (0.86–0.93) | 0.85 (0.82–0.89) | 0.88 (0.84–0.92) | 0.86 (0.82–0.89) |
|  | △NIHSS 24h | 0.85 (0.80–0.90) | 0.78 (0.74–0.83) | 0.82 (0.76–0.87) | 0.79 (0.74–0.84) |
|  | NIHSS percent change | 0.85 (0.80–0.90) | 0.78 (0.73–0.82) | 0.82 (0.76–0.87) | 0.78 (0.73–0.83) |
|  | cENI | 0.60 (0.54–0.66) | 0.60 (0.54–0.65) | 0.60 (0.54–0.65) | 0.58 (0.53–0.64) |
|  | dENI | 0.77 (0.71–0.83) | 0.67 (0.62–0.73) | 0.75 (0.69–0.81) | 0.70 (0.64–0.76) |
|  | mENI | 0.76 (0.70–0.83) | 0.66 (0.61–0.72) | 0.74 (0.68–0.80) | 0.69 (0.63–0.75) |


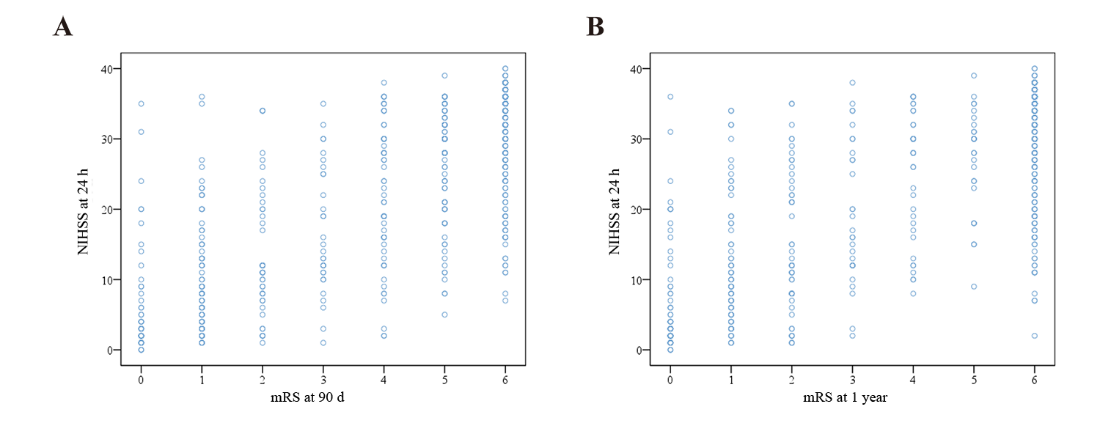


**eFigure 1.** (A-B) Scatter plot showing the association between 24-h NIHSS and modified Rankin Scale (mRS) at 90 days and 1 year.


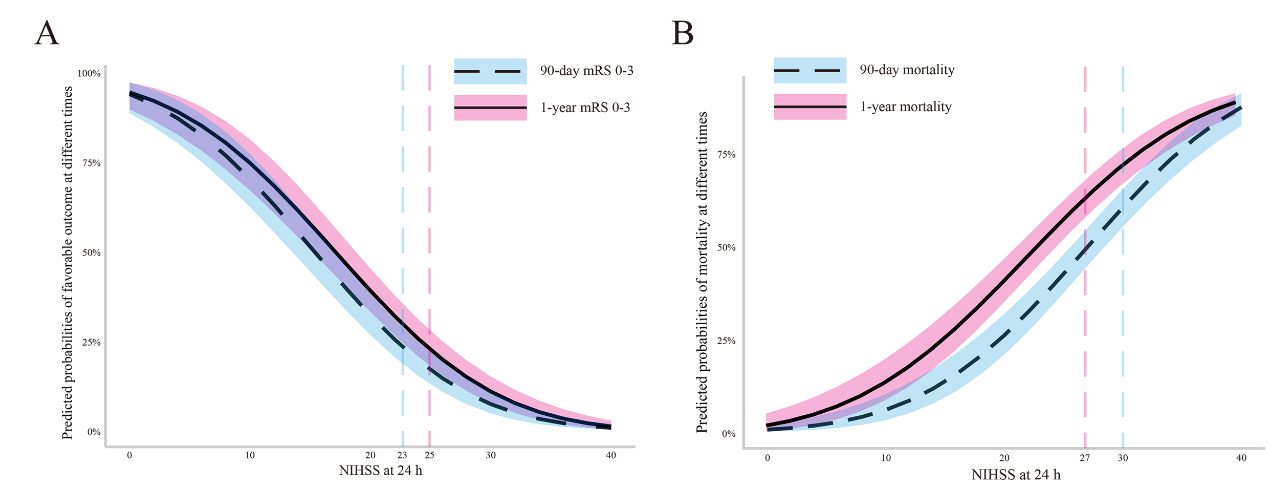


**eFigure 2.** Predicted probability of short and long-term clinical outcomes according to 24-h NIHSS. (A)Predicted probability of favourable outcome at 90 days and 1 year. (B) Predicted probability of mortality at 90 days and 1 year.
